# Resilience and coping strategies of undergraduate medical students at the University of the Free State

**DOI:** 10.4102/sajpsychiatry.v26i0.1471

**Published:** 2020-07-28

**Authors:** Lynette J. van der Merwe, Anja Botha, Gina Joubert

**Affiliations:** 1 Undergraduate Medical Programme Management, School of Clinical Medicine, Faculty of Health Sciences, University of the Free State, Bloemfontein, South Africa; 2 School of Clinical Medicine, Faculty of Health Sciences, University of the Free State, Bloemfontein, South Africa; 3 Department of Biostatistics, Faculty of Health Sciences, University of the Free State, Bloemfontein, South Africa

**Keywords:** medical studies, resilience, coping skills, burnout, stress factors

## Abstract

**Background:**

Medical studies place students at risk for burnout. Resilience enables students to cope with adversity. Students’ coping skills will ensure the well-being of future healthcare professisonals.

**Objectives:**

This study investigated resilience and coping among undergraduate medical students.

**Setting:**

Undergraduate students at the University of the Free State medical school.

**Methods:**

A cross-sectional study was performed. Quantitative data regarding resilience (Connor-Davidson Resilience Scale), coping strategies (Brief COPE questionnaire) and relevant information were collected by means of an anonymous self-administered questionnaire.

**Results:**

Five hundred students (pre-clinical *n* = 270; clinical *n* = 230; approximately 62% female) participated. Most students self-reported high resilience (84.6% pre-clinical; 91.8% clinical). Mean resilience scores were 72.5 (pre-clinical) and 75.4 (clinical). Clinical students had higher resilience scores, while black, pre-clinical, first-generation and female students scored lower.

Academic stress was most prominent (> 85%) and associated with lower resilience scores. Most students used adaptive coping strategies (e.g. instrumental or emotional support) associated with significantly increased resilience scores. Students who used dysfunctional strategies (e.g. substance abuse) had significantly lower resilience scores.

**Conclusion:**

Associations between resilience scores and year of study, gender, ethnicity, levels and type of stress varied. Academic pressure was a major source of stress. Adaptive coping strategies were associated with higher resilience scores.

## Introduction

Medical studies place numerous demands on students, which continue into the future physician’s career, making both students and healthcare workers vulnerable to burnout.^1,2,3,4^ Successful selection for medical school is merely the first hurdle in an increasingly complex, competitive and challenging arena. Demanding academic requirements as well as assessment and performance pressure, personal and financial adversity, early clinical exposure and the challenge of dealing with life and death issues, tough working environments and public expectations contribute to the rising rate of burnout.^5^ This may result in suicidal ideation, substance abuse, poor academic performance, dishonesty or dropout, professional lapses and poor clinical competence.^1,2,6^

Burnout as an occupational phenomenon described in the revised ICD-11 is ‘a syndrome conceptualised as resulting from chronic workplace stress that has not been successfully managed’. It includes three dimensions, namely ‘feelings of energy depletion or exhaustion’, ‘increased mental distance, or feelings of negativism or cynicism related to one’s job’ and ‘reduced professional efficacy’.^7^

Resilience is described as the ability to bounce back, grow stronger from failure, and remain positive in the midst of hard times.^8^ It may be regarded as ‘the other leg to stand on’,^9^ enabling physicians and students to cope and thrive despite adversity. It is a mindset including skills and attributes that can be learned, developed and nurtured, and is not necessarily just an individual trait.^10^ Various interventions have been proposed to foster resilience, including mindfulness training, Balint groups, guided reflection and mentoring.^1,11^ However, training programmes to develop resilience have not yet proven to offer the optimal solution for the prevention of student burnout.^1,12^

In a small study among 94 medical students at the University of KwaZulu-Natal, South Africa, lifestyle changes and constructive engagement with various support structures were most commonly used. This study reported that at least 78% of students experienced stress during their medical studies.^13^ In a recent study at the University of Cape Town’s Faculty of Health Sciences, almost a quarter of medical students had diagnoses of depression or anxiety, requiring psychotropic medication. This study highlighted the significantly higher risk among medical students to suffer from these disorders and emphasised the need for strengthening mental well-being and addressing institutional mental health culture.^14^

In the face of concern about the wellness of the future physician workforce, it may be valuable to uncover coping strategies among resilient students. Identifying risks and protective factors can provide a more nuanced view of resilience as a competence. This ‘deconstruction’ of resilience, probing deeper than merely offering it as a panacea for burnout,^4^ will guide future planning of educational approaches aimed at promoting student well-being.^3^ This includes a focus on coping behaviours^10^ that may alleviate or exacerbate the problem.

The aim of this study was to obtain a greater understanding of resilience and coping behaviours and the associations between resilience, coping strategies and demographic factors among undergraduate medical students, in order to inform intervention programmes focusing on prevention of burnout and enhancing resilience.

## Methods

This study formed part of a larger research project on resilience in undergraduate medical students at the University of the Free State (UFS). For the purpose of this article, we collected quantitative data regarding resilience, coping strategies and associated factors by means of a standardised, validated anonymous self-administered questionnaire that also obtained demographic data.

The target population included all students in the undergraduate medical programme at the UFS Faculty of Health Sciences, who were invited to participate in a questionnaire survey. All undergraduate students registered in the five-year MB ChB programme, from first to final year, who were at least 18 years of age at the time of data collection, were eligible to participate.

The five-year programme is divided into three phases and 10 semesters. Students in Phase I (Semester 1) and II (Semesters 2–5) are pre-clinical students, and those in Phase III (Semesters 6–10) are clinical students. The study was done in the second semester of the academic year. Students in Semesters 2 and 4 (pre-clinical) and Semesters 6, 8 and 10 (clinical) were invited to participate in the study.

The questionnaire included variables such as demographic information (age, gender, race, year of study, relationship status), perceptions regarding students’ own level of resilience, as well as the Connor-Davidson Resilience Scale (CDR)^15^ and Brief COPE questionnaire.

The CDR^15^ was used to determine the students’ resilience. It comprises 25 items rated on a five-point Likert scale with responses ranging from ‘Not true at all’ to ‘True nearly all of the time’. The total score ranges from 0 to 100, with higher scores indicating higher resilience. Connor and Davidson^15^ reported an alpha coefficient of 0.87 for a group of adults struggling with anxiety. Jørgensen and Seedat report an alpha coefficient of 0.93 for a diverse group of South African adolescents. They recommend this instrument as useful for measuring resilience in the South African population.^16^ More recently, the instrument was used for a study among South African adults (comparing a clinical population with a non-clinical population) and the reliability coefficient was reported to be 0.94.^17^

The Brief COPE^18^ measures the following coping styles: (1) self-distraction, (2) active coping, (3) denial, (4) substance use, (5) use of emotional support, (6) use of instrumental support, (7) behavioural disengagement, (8) venting, (9) positive reframing, (10) planning, (11) humour, (12) acceptance, (13) religion and (14) self-blame. It consists of 28 items rated on a four-point Likert scale with responses ranging from ‘I haven’t been doing this at all’ to ‘I have been doing this a lot’. A total score is not calculated. The subscales are calculated separately resulting in 14 continuous variables. The minimum (2) and maximum (8) scores for each subscale indicate low and high reliance on the coping skill. The scales are used individually to obtain a profile of the participant’s coping style. The alpha coefficients for the various subscales vary between 0.50 and 0.90 for an American adult sample.^18^ In South Africa, alpha coefficients of 0.63 (in a sample of HIV-positive women) and 0.77 (in a sample of students) have been reported.^19^

In addition to anonymous completion of the questionnaire, data were managed with strict confidentiality. One of the co-authors (G.J.) in the Department of Biostatistics performed the statistical analysis of quantitative data, using SAS software (SAS Institute; Cary, NC, USA). The analysis included the calculation of descriptive statistics and reliability coefficients, as well as the associations between resilience scores, demographic data and other factors affecting resilience. Numerical variables of subgroups were compared using *t*-tests and analysis of variance (ANOVA), and 95% confidence intervals were calculated for differences in means.

A pilot study was done on five students in the undergraduate Nutrition and Dietetics programme to ensure that the time taken to complete the questionnaire was sufficient, and to determine any logistical issues related to completion of the questionnaire. The data from the pilot study were not included in the analysis.

The questionnaires were distributed during classroom contact sessions to all undergraduate medical students in the MB ChB programme by one of the researchers. Students were informed that participation was voluntary and anonymous, and could ask questions to clarify concepts. An information document was provided to all participants explaining the study.

Students were advised that they would be referred to a clinical psychologist for support if they experienced any distress because of completion of the questionnaire, and were given contact details of a psychologist.

### Ethical consideration

The Health Sciences Research Ethics Committee of the University of the Free State granted approval to conduct the study (HSREC 63/2017). The relevant university authorities gave permission to include the students in the research.

## Results

A total of 500 from the sample population of 696 (71.8%) students completed the questionnaires. They were divided into pre-clinical (*n* = 270, 54.0%; response rate 79.2%; year 1 and 2; mean age 20 years) and clinical (*n* = 230, 46.0%; response rate 62%; year 3, 4 and 5; mean age 22.6 years) groups. The participants included 166 (63.9%) female students in the pre-clinical and 127 (57.2%) in the clinical groups. Only 55 (20.5%) of pre-clinical and 34 (15.6%) of clinical students reported that they were first-generation students (defined in this study as students whose parents had never been enrolled in post-school higher education).

Table 1 shows the resilience scores of pre-clinical and clinical students, determined by means of the CDR.

**TABLE 1:** Resilience scores and associated factors of pre-clinical and clinical medical students.

Variable	Pre-clinical students					Clinical students				
	*n*/N	%	Mean CDR	SD	ANOVA/ *t*-test *p*-value	*n*/N	%	Mean CDR	SD	ANOVA/ *t*-test *p*-value
**Perceived level of resilience**										
High	220/260	84.6	75.3	12.5	< 0.01	201/219	91.8	77.1	10.3	< 0.01
Low	40/260	15.4	57.7	16.0		18/219	8.2	57.5	11.2	
**Gender**										
Male	94/260	36.2	74.9	12.7	0.24	95/222	42.8	77.4	11.4	0.03
Female	166/260	63.9	71.7	15.5		127/222	57.2	73.9	11.8	
**First-generation student**										
Yes	55/269	20.5	71.0	13.9	0.41	34/218	15.6	75	9.4	0.93
No	214/269	79.6	72.9	14.7		184/218	84.4	75.2	12.0	
**Ethnicity**										
Black	119/207	57.5	70.8	14.7	< 0.01	23/192	12.0	76.3	14.8	0.81
White	88/207	42.5	77.0	13.6		169/192	88.0	75.7	11.1	
**Current degree of stress**										
None/minor	94/260	36.2	77.4	11.2	< 0.01	57/221	25.8	80.3	11.0	< 0.01
Mild	131/260	50.4	70.8	15.6		118/221	53.4	75	10.3	
Severe/devastating	35/260	13.5	65.2	13.9		47/221	21.3	70.3	13.6	
**Academic stress**										
No	31/256	12.1	78.1	11.2	0.02	30/219	13.7	80.6	13.1	0.01
Yes	225/256	87.9	71.5	14.8		189/219	86.3	74.4	11.3	
**Personal stress**										
No	130/256	50.8	75.2	12.6	< 0.01	102/219	46.6	76.8	11.2	0.07
Yes	126/256	49.2	76.8	15.8		117/219	53.4	73.9	12.0	
**Financial stress**										
No	169/256	66	73.3	14.8	0.13	153/219	69.9	75	11.8	0.64
Yes	87/256	34.0	70.4	14		66/219	30.1	75.8	11.6	
**Other stressors**										
No	244/256	95.3	72.2	14.7	0.46	213/219	97.3	75	11.7	0.07
Yes	12/256	4.7	75.3	11.4		6	2.7	83.8	10.0	
**No stressors**										
No	250/256	97.7	72.1	14.5	0.18	206/219	94.1	74.8	11.4	0.02
Yes	6/256	2.3	80.2	14.9		13/219	5.9	82.9	14.8	

CDR, Connor-Davidson Resilience Scale.

Overall, in the pre-clinical group, the mean score obtained on the CDR was 72.5 (standard deviation [SD] 14.5), while clinical students scored significantly higher (75.4; SD 11.7; *p* = 0.02; 95% CI 0.6; 5.2). The mean scores on the CDR for pre-clinical (84.6%) and clinical (90.5%) students who perceived themselves as highly resilient were 75.3 (SD 12.5) and 77.1 (SD 10.3), while the mean scores for students who reported perceived low resilience were 57.7 (SD 16.0) and 57.5 (SD 11.2) for the pre-clinical and clinical groups, accordingly. These differences were statistically significant (*p* < 0.01). The Cronbach’s alpha values determined for the pre-clinical and clinical groups were 0.92 and 0.91.

In both pre-clinical and clinical groups, there were no significant differences in resilience scores between male and female students, although male students in both groups had higher scores on the CDR. Although first-generation students scored lower on the CDR, these differences were not statistically significant. When comparing ethnic groups, black pre-clinical students had significantly lower resilience scores than white students, which was not the case in the clinical group where white students had slightly lower, although not significant, CDR scores.

More than a third of pre-clinical (*n* = 94, 36.2%) and a quarter of clinical students (*n* = 57, 25.8%) reported no or minor stress experienced at the time of the study. These students had significantly higher CDR scores in both the pre-clinical and clinical groups, and these scores were progressively and significantly lower as students reported their current degree of stress as mild to severe or devastating. Students who reported academic stress (pre-clinical *n* = 225, 87.9%; clinical *n* = 189, 86.3%) had significantly lower CDR scores in both groups. Pre-clinical students who reported personal stress (*n* = 126, 49.2%) had significantly lower CDR scores, while the slightly higher percentage of clinical students who reported personal stress (*n* = 117, 53.4%) did not have significantly lower CDR scores. There were no significant differences in CDR scores in both groups of students who reported financial (pre-clinical *n* = 87, 34.0%; clinical *n* = 66, 30.1%) or other (pre-clinical *n* = 12, 4.7%; clinical *n* = 6, 2.7%) stressors. In the clinical group, CDR scores were significantly higher in students who reported no stressors (*n* = 13, 5.9%), but these numbers were very low.

In Table 2, the results of the Brief COPE questionnaire in 14 coping styles are compared between pre-clinical and clinical students, and the associations with resilience scores obtained on the CDR are shown. The Brief COPE scores are indicated as 2–3 (low reliance), 4–6 (average) and 7–8 (high reliance) for each of the coping styles.

**TABLE 2:** Association between the Brief COPE questionnaire and Connor-Davidson Resilience scale scores for pre-clinical and clinical students.

Variable	Pre-clinical students					Clinical students				
	Brief COPE		Mean CDR	SD	ANOVA *p*-value	Brief COPE		Mean CDR	SD	ANOVA *p*-value
	Median (range)					Median (range)				
	*n*/N	%	*n*/N			%				
**Self-distraction**	5	2–8	-	-		5	2–8	-	-	0.98
Score 2–3	40/258	15.5	73.2	14.3	0.16	27/221	12.2	81.2	11.5	0.02*
Score 4–6	159/258	61.6	71.4	14.4		153/221	69.2	74.8	11.2	
Score 7–8	59/258	22.9	75.6	14.2		41/221	18.6	73.8	12.9	
**Active coping**	6	2–8	-	-		6	2–8	-	-	0.40
Score 2–3	18/259	6.9	61.8	14.4	< 0.01*	22/221	10	71.4	14.7	< 0.01*
Score 4–6	162/259	62.6	70.6	14.9		137/221	62	74.2	11.5	
Score 7–8	79/259	30.5	78.6	11.2		62/221	28	79.6	10.0	
**Denial**	2	2–8	-	-		2	2–7	-	-	0.01
Score 2–3	212/259	81.9	73.3	13.7	0.05	195/221	88.2	75.7	11.3	0.38
Score 4–6	43/259	16.6	67.6	17.8		26/221	11.8	73.4	14.9	
Score 7–8	4/259	1.5	77.3	7.2		0	0	–	-	
**Substance use**	N/A†	N/A†	N/A†	N/A†	N/A†	2	2–8	-	-	N/A†
Score 2–3						173/220	77.3	76.6	12.0	0.01*
Score 4–6						40/220	18.2	70.7	9.3	
Score 7–8						7/220	3.2	71.1	10.5	
**Emotional support**	5	2–8	-	-		6	2–8	-	-	< 0.01*
Score 2–3	48/260	18.5	69.2	16.4	< 0.01*	22/221	10.0	70.1	13.5	0.06
Score 4–6	144/260	55.4	71.2	14.8		122/221	55.2	75.5	11.3	
Score 7–8	68/260	26.1	77.6	11.0		77/221	34.8	76.8	11.6	
**Behavioural disengagement**	2	2–8	-	-		2	2–8	-	-	0.87
Score 2–3	180/258	69.8	75.8	12.8	< 0.01*	155/221	70.1	77.8	10.6	0.09
Score 4–6	75/258	29.1	65.2	15.0		62/221	28.1	69.9	11	
Score 7–8	3/258	1.1	55.7	29.7		4/221	1.8	69	30.3	
**Venting**	4	2–8	-	-		5	2–8	-	-	0.51
Score 2–3	72/260	27.2	73.5	15.2	0.58	56/220	25.5	75.1	13.1	0.70
Score 4–6	163/260	62.7	71.8	14.3		144/220	65.5	75.8	11.1	
Score 7–8	25/260	9.6	74.4	13.8		20/220	9.0	73.5	12.5	
**Instrumental support**	5	2–8	-	-		5	2–8	-	-	0.90
Score 2–3	51/259	19.7	67.3	16.7	< 0.01*	34/221	15.4	72.7	10.8	0.03*
Score 4–6	144/259	55.6	71.8	14.6		135/221	61.1	74.7	12.0	
Score 7–8	64/259	24.7	78	10.3		52/221	23.5	79.0	11.2	
**Positive reframing**	6	2–8	-			6	2–8	-	-	0.81
Score 2–3	23/260	8.9	63.3	15.9	< 0.01*	15/221	6.8	64.7	17.9	< 0.01*
Score 4–6	148/260	56.9	69.4	13.8		138/221	62.4	73.8	10.5	
Score 7–8	89/260	34.2	80.1	12.1		68/221	30.8	81.2	9.7	
**Self-blame**	5	2–8	-	-		4	2–8	-	-	0.09
Score 2–3	76/260	29.2	77.6	12.1	< 0.01*	80/221	36.2	78.9	10.7	< 0.01*
Score 4–6	137/260	52.7	71.7	13.4		113/221	51.1	74.1	11.0	
Score 7–8	47/260	18.1	66.8	18.5		28/221	12.7	70.8	14.8	
**Planning**	6	2–8	-			6	2–8	-	-	0.03*
Score 2–3	20/260	7.7	65.8	17.2	0.01*	19/220	8.6	73.9	13.0	0.10
Score 4–6	160/260	61.5	70.2	14.4		152/220	69.1	74.5	11.9	
Score 7–8	80/260	30.8	78.8	11.9		49/220	22.3	78.5	10.4	
**Humour**	5	2–8	-	-		5	2–8	-	-	0.22
Score 2–3	85/260	32.7	69.5	14.7	0.01*	51/221	23.1	75.7	12.7	0.28
Score 4–6	119/260	45.8	72.7	15.3		120/221	54.2	74.8	11.6	
Score 7–8	56/260	21.5	76.9	11.4		50/221	22.6	77.7	11.0	
**Acceptance**	N/A†	N/A†	N/A†	N/A†	N/A†	6	2–8	-	-	N/A†
Score 2–3						8/220	3.6	81.0	9.4	0.01*
Score 4–6						144/220	65.5	73.7	12.0	
Score 7–8						68/220	30.9	78.2	10.8	
**Religious coping**	7	2–8	-	-	-	6	2–8	-	-	< 0.01*
Score 2–3	27/260	10.4	59.9	15.2	< 0.01*	36/221	16.3	71.2	11.4	< 0.01*
Score 4–6	87/260	33.5	69.7	14.3		88/221	39.8	73.1	12.4	
Score 7–8	146/260	56.1	76.6	12.8		97/221	43.9	79.1	10.2	

* , Statistically significant difference.

† , Pre-clinical students did not complete these items on the Brief COPE questionnaire.

N/A, not applicable.

Pre-clinical students did not complete the questions related to substance abuse and acceptance; therefore, no comparisons were possible between the two groups regarding this coping style. Pre-clinical students had significantly higher reliance on religious coping than clinical students, while clinical students had significantly higher reliance on emotional support than pre-clinical students. There were no significant differences between pre-clinical and clinical students regarding the other coping styles.

The coping styles that were associated with significantly higher CDR scores in both the pre-clinical and clinical groups included active coping, instrumental support, positive reframing and religious coping. The majority of both pre-clinical and clinical students had average to high reliance on these coping styles. In pre-clinical students, the majority of whom had average to high reliance on emotional support, planning and humour as coping strategies, significantly higher CDR scores were found when these coping styles were used.

In clinical students, the majority had average to high reliance on acceptance as a coping strategy, which was associated with significantly higher CDR scores. In the students who had low reliance on this coping style with an associated significantly higher CDR score, the finding should be interpreted with caution due to the low number (*n* = 8). Clinical students (*n* = 68, 30.9%) who had high reliance on acceptance as coping style had significantly higher CDR scores than those with average reliance on this coping style (*n* = 144, 65.5%).

In both groups, average to high reliance on self-blame was associated with significantly lower CDR scores.

In clinical students, average to high reliance on substance abuse and self-distraction was associated with significantly lower CDR scores. A small percentage of clinical students had average (*n* = 40, 18.2%) or high (*n* = 7, 3.2%) reliance on substance abuse as a coping style, while the majority of these students had average (*n* = 153, 69.2%) to high (*n* = 41, 18.6%) reliance on self-distraction as a coping style. The majority of pre-clinical students showed low reliance on behavioural disengagement as a coping style, which was associated with significantly higher CDR scores.

## Discussion

This study addressed the need to explore medical students’ resilience strategies.^5^ Studies among South African medical students have shown a significantly increased risk of major depression or anxiety diagnoses.^13,14^ Among a group of pre-clinical and clinical undergraduate medical students, the majority of students self-reported high resilience, and scored significantly higher on the CDR, pointing to good self-awareness.

Factors that promote or protect against burnout include personal characteristics (including demographic variables), perceptions of the learning environment, social support and levels of stress.^20^ We found that the year of study, ethnicity, gender and parental educational status influenced resilience scores. Clinical students (years 3, 4 and 5) had significantly higher CDR scores than pre-clinical students (years 1 and 2), which could indicate an increase in personal development as their studies progressed. Black pre-clinical students had significantly lower resilience scores, while white clinical students’ CDR scores were lower, although not significantly different, than the scores obtained by students of other ethnic groups. Historical disadvantage may influence resilience, especially among black students, and this is illustrated by the fact that first-generation students also had lower CDR scores, although not significant. Male students had higher CDR scores, but these were not significantly different from female students’ scores. This supports the reported association between female gender and depressive or anxiety disorders.^14^

We found that the CDR score was significantly lower in both pre-clinical and clinical students who reported a current high degree of stress. Stress is inherent to medical studies, but also a common risk factor for burnout.^21^ The fact that most students self-reported high resilience, a mild degree of stress, and scored high on the CDR, might point to effective coping strategies among this cohort of medical students.

Academic stress was most prominent, and the majority of students indicated that they experienced this stressor, which was associated with lower CDR scores, although not significantly. Whether academic stress among this cohort of medical students is higher than that of other students enrolled in tertiary studies should be investigated further.

Approximately half of both groups of students reported personal stress. Of note was that pre-clinical students who reported no personal stress had significantly lower CDR scores, which was surprising and might need further investigation. In clinical students, the presence of personal stress was associated with slightly lower CDR scores, but not significantly. Senior students’ ability to manage adversity better in their personal lives should be investigated further.

Approximately one-third of students reported financial stress, but no significant association with CDR scores was observed. This finding was also surprising considering the fact that most South African students at tertiary institutions experience financial need, as reported in the South African Surveys of Student Engagement Annual Report 2016.^22^ It might be that students in health sciences faculties or health professions programmes have more access to bursaries and scholarships compared to students in other faculties.

What factors contributed to this cohort of students’ resilience? Coping refers to the individual’s cognitive and behavioural strategies to deal with perceived stressors, and may be functional or dysfunctional with a respective increased or decreased probability of burnout.^3^ Coping strategies have also been described as approach-oriented, positive or adaptive (associated with lower risk burnout and improved resilience) or avoidant, maladaptive or negative (associated with higher risk for burnout and decreased resilience).^6,9^

For example, Erschens et al.^3^ found that functional coping strategies in a population of 597 medical students in Germany included seeking support from friends or family, doing relaxing exercise, doing sports and being involved with fellow students. They found a negative association with burnout among students who displayed these behaviours, in contrast to those who made use of dysfunctional coping strategies, including taking tranquilizers, stimulants or alcohol, ruminating and playing games on a personal computer or mobile phone.^3^

Similarly, Thompson et al.^6^ found among 161 medical students that approach-oriented (positive) coping strategies (e.g. exercise, support from family, friends or counsellors) put them at lower risk for burnout, while avoidant coping strategies were associated with more mental health issues.

In this study, we found that CDR scores were significantly higher in both pre-clinical and clinical students who used coping styles that included active coping, instrumental support, positive reframing and religious coping, indicating these as functional or adaptive coping styles. Among pre-clinical students, emotional support, planning and humour, and in clinical students, acceptance, were additional functional or adaptive coping styles, associated with significantly higher CDR scores.

Interventions aimed at fostering and developing resilience on both individual and system-wide levels, need to be researched further.^1^ When planning interventions or support for medical students to enhance resilience, it would be helpful to focus on these coping styles and consider the role of personal factors that may increase resilience.^6^ For example, active coping involves the steps an individual could take to improve the situation in which they find themselves. Instrumental support refers to getting help and advice from others, and positive reframing would mean seeing circumstances in a more positive light or finding the good in a situation.

Religious coping refers to the individual finding comfort in religion or spiritual beliefs or engaging in practices such as praying or meditating. In addition, irrespective of the situation they find themselves in, individuals may learn or develop the ability to get emotional support, comfort and understanding from others, plan or strategise the next move, and use humour to deal with or accept situations. These coping mechanisms are congruent with previously published research linking the beliefs of the individual’s ability to positively impact on their environment, and that continuous effort is worthwhile despite adversity.^10^

Our findings are supported by literature indicating that social support reduces the probability of suffering from stress and exhaustion, and is important for nurturing resilience in medical students,^20^ and that sports and relaxation techniques are helpful in managing stress.^3,5^ This includes a focus on the learning environment and reducing stress.^2^ In other words, interventions should focus on individual and system levels to empower students to engage in activities such as sport and relaxation, as well as obtaining support from family, friends and peers.

In both pre-clinical and clinical students in our study, self-blame (being critical of oneself), self-distraction (turning to work, entertainment or daydreaming) and substance abuse among clinical students, or behavioural disengagement (giving up) among pre-clinical students, could be regarded as maladaptive and dysfunctional coping styles, as these were associated with significantly lower CDR scores. Erschens et al.^3^ and Thompson et al.^6^ also described these behaviours, pointing towards the need to create awareness about the negative impact of these coping strategies, and to empower students to choose healthier options to prevent burnout and develop resilience.

## Strengths and limitations

A strength of this study was that it provided a cross-sectional view of a large population of students from first to final year of an undergraduate medical programme, with fair to good response rates. It not only focused on individual constructs (resilience, stress and coping strategies), but also determined the associations between these constructs, providing a comprehensive exploration of what constitutes resilience in a cohort of medical students. Validated questionnaires were used, providing data that may be compared with findings from other populations.

The study had a number of limitations. Firstly, the cross-sectional nature of the study implied that the associations could not be used to identify causal relationships, and the consequences of coping strategies should be viewed accordingly. Secondly, the timing in the academic year could have meant that students’ resilience was over-estimated and their stress underestimated. The survey took place during the third quarter of the academic year when no final assessments were scheduled. Thirdly, because students’ self-reported data were used, the findings might have been misleading, as students’ responses could have been influenced by stigma associated with mental health issues.^6^

## Conclusion

In this cohort of first-year to final-year undergraduate medical students at the University of the Free State, the majority of students had self-reported high resilience associated with high resilience scores using the CDR. Clinical students scored significantly higher than pre-clinical students, while black pre-clinical students had significantly lower resilience scores. Female and first-generation students had slightly lower resilience scores, although not significant. Academic stress was most commonly reported and associated with lower resilience. Fewer students reported personal or financial stress that was not associated with lower resilience scores. In general, most students used adaptive coping strategies that were associated with significantly higher resilience. These include active coping, instrumental support, positive reframing, religious coping, emotional support and acceptance. Although very few students relied on mal-adaptive coping strategies, including self-blame, self-distraction or substance abuse, these were associated with significantly lower resilience scores.

Interventions to nurture resilience in medical students should focus on both individual and systems levels to empower students to engage in functional, positive strategies cultivating resilience. In the context of the UFS Faculty of Health Sciences, this could include providing workshops for students on mechanisms to develop adaptive coping strategies. Further research could focus on investigating what other forms of support students would require besides existing structures, such as academic support through the Division Student Learning and Development, as well as psycho-social support through a clinical psychologist and social worker.
